# Hypnotism for the Insane

**Published:** 1893-01-28

**Authors:** 


### CONTEMPORARY THEORY AND RESEARCH.

### HYPNOTISM FOR THE INSANE.

THE "Journal of Mental Science" has a remarkable article on this subject, by Dr. Robertson, of the Edinburgh Asylum. In his own employment of hypnotism among his patients, Dr. Robertson has found it of essential service in the following particulars. First, and perhaps most important, in inducing sleep where often every kind of sedative has been tried in vain. Where the mania is too acute the hypnotism has occasionally failed, but cases are mentioned where the pressure of the hand has sufficed to send the patient to sleep, in spite of the most determined resistance, even to closing the eyes. As a sedative in states of excitement and insubordination, hypnotism has proved most valuable, having frequently acted as a substitute for restraint by calming the patient into submission. While sending the patient to sleep, Dr. Robertson is in the habit of making suggestions to him, which being impressed on the brain while it is in an abnormally sensitive condition, are invariably carried out on awaking. Thus a patient who has developed a morbid resistance to taking food or medicine, will swallow either without difficulty if he has been previously told by the hypnotiser that he will do so. As these hypnotic suggestions are carried out very literally by the patient, great caution is necessary in making them. Dr. Robertson quotes a case in which he assured a patient, who fancied herself crippled by rheumatism, that she would walk the length of the ward on awaking. She carried out his instructions a little too faithfully, and was not to be restrained from pacing the ward during the entire afternoon and evening, to the considerable annoyance of the nurses and other patients. Dr. Robertson's experiences demand consideration.

